# Morphological, Phytochemical, and Molecular Characterization of Advanced Tomato Lines Under Heat Stress Conditions

**DOI:** 10.1002/pei3.70220

**Published:** 2026-10-09

**Authors:** Haidar Anwar Arkwazee, Salam Mahmud Sulaiman, Kamaran Salh Rasul, Hawar Halshoy, Shwana Ahmed Braim

**Affiliations:** ^1^ Horticulture and Landscape Design Department, College of Agricultural Engineering Sciences University of Sulaimani Sulaymaniyah Kurdistan Region Iraq; ^2^ Chemistry and Forensics Department, School of Science and Technology Nottingham Trent University Nottingham UK

**Keywords:** antioxidant activity, genetic variability, heat stress tolerance, proline accumulation, *Solanum lycopersicum*, tomato genotypes, yield components

## Abstract

Heat stress severely limits tomato growth, yield, and biochemical stability, highlighting the need for thermotolerant genotypes. This study evaluated six advanced tomato lines using morphological, biochemical, genetic, and molecular analyses under heat stress conditions. Significant genotypic differences (*p* < 0.001) were observed across the evaluated traits, with TAL101‐2 and TAL101‐1 exhibiting better growth and reproductive performance under high‐temperature conditions. TAL105 showed better growth and antioxidant activity, whereas TAL104 exhibited strong biochemical adaptation, as evidenced by elevated phenolic content and antioxidant activity. TAL101‐2 maintained relatively high fruit set despite limited vegetative growth. High heritability and genetic advance for reproductive and phytochemical traits indicated strong genetic control. Correlation and principal component analyses revealed positive relationships between vegetative vigor and yield, whereas biochemical traits were associated with oxidative stress tolerance. Molecular and phylogenetic analyses confirmed clear genetic diversity among the lines. Stress‐responsive gene profiling also revealed distinct marker activity patterns. TAL105 and TAL104 were identified as promising candidates for breeding heat‐tolerant tomato cultivars.

## 1 Introduction

Tomato ( *Solanum lycopersicum* ) is one of the most widely cultivated and economically important vegetable crops worldwide, valued for its high nutritional content, particularly lycopene and vitamins, its culinary versatility, and its significant contributions to the global agricultural economy (Halshoy 2025; Zhou et al. 2015). Global tomato production reached approximately 186 million metric tons in 2024 (Sánchez et al. 2024), with major producers including China, Mexico, Italy, Spain, and the USA (Karachun and Lebedynskyi 2024), supplying both fresh markets and processing industries. Beyond its economic importance, the tomato plays a critical role in food security and farmers' livelihoods in many regions, including Iraq and particularly the Kurdistan region, where it is extensively cultivated under plastic‐house conditions to enhance productivity and extend the growing season (Mohammed et al. 2023; Tegen et al. 2016).

Despite its global importance, tomato production is increasingly threatened by rising temperatures associated with climate change that frequently exceed the optimal range for normal plant growth and development (John and Stephen 2024). High‐temperature stress disrupts key physiological processes, including photosynthesis, transpiration, and pollen viability, ultimately reducing fruit set and causing significant yield losses (Graci et al. 2023; Mehmood et al. 2025). Temperatures above 30°C during the flowering stage are particularly detrimental, causing flower abscission and impaired fertilization. These effects are intensified in arid and semi‐arid regions, where heat stress has become a major constraint to sustainable tomato production and agricultural stability (Hoshikawa et al. 2021; Mohammed et al. 2025).

Addressing heat stress in tomato requires a multifaceted approach; however, genetic improvement remains the most effective and sustainable long‐term solution. Breeding for thermotolerance focuses on traits associated with growth stability, reproductive success, and physiological resilience, including proline accumulation, antioxidant activity, and maintenance of cellular integrity (Bollier et al. 2023; Khan et al. 2024). Agronomic strategies such as optimized plant spacing, canopy management, and microclimate regulation through ventilation and shading also help mitigate heat stress (Ro et al. 2021). Additionally, the application of osmoprotectants and plant growth regulators, such as proline and gibberellic acid (GA_3_), has been shown to enhance stress tolerance by stabilizing photosynthesis and strengthening antioxidant defense systems (de la Torre‐González et al. 2018; Guo et al. 2022; Shah Jahan et al. 2019). However, the effectiveness of these approaches is often genotype‐dependent, reflecting plants' inherent genetic capacity to regulate physiological and biochemical responses under heat stress.

Genotypic variation plays a central role in determining heat tolerance, as different tomato lines exhibit varying abilities to maintain growth, reproductive performance, and cellular stability under elevated temperatures (Fernández‐Crespo et al. 2022). Some genotypes achieve tolerance through morphological vigor and sustained assimilate production, while others rely more heavily on biochemical defense mechanisms, including the accumulation of osmoprotectants and antioxidant compounds (Akram et al. 2024; Lee et al. 2022). Understanding how these traits interact to influence overall plant performance is essential for effective selection and breeding. In this context, multivariate approaches such as correlation analysis and principal component analysis (PCA) provide powerful tools to unravel complex trait relationships and identify key determinants of thermotolerance (Joe et al. 2026; Singh et al. 2022).

In addition to phenotypic and biochemical evaluations, molecular characterization provides critical insight into the genetic basis of stress responses. Using stress‐related molecular markers, particularly those associated with transcriptional regulation and stress signaling pathways, enables the identification of genotypes with enhanced adaptive capacity. Integrating molecular data with morphological and biochemical traits yields a more comprehensive understanding of thermotolerance, bridging the gap between observed plant performance and underlying genetic mechanisms. By linking multivariate trait analysis with molecular validation, this research provides a robust framework for developing heat‐tolerant tomato cultivars adapted to high‐temperature environments, such as those prevailing in Kurdistan and similar agroecological regions (Haakim et al. 2025).

This study is therefore designed to evaluate newly developed advanced tomato lines under chronic heat stress conditions in plastic‐house environments in the Kurdistan region. A holistic approach was adopted, encompassing morphological traits (e.g., plant height, branching, and cluster formation), reproductive yield components (e.g., fruit number and fruit set), and key biochemical indicators of stress response, including proline content, antioxidant activity, and lipid peroxidation (MDA levels) (Najafi et al. 2025; Rodrigues 2021). Furthermore, molecular analysis using stress‐related markers was conducted to elucidate the genetic relationships among the evaluated lines and to support the interpretation of phenotypic and biochemical responses. This study aims to characterize the integrated responses of advanced tomato lines to heat stress and identify genotypes exhibiting superior performance at the morphological, biochemical, and molecular levels, thereby providing reliable selection criteria for breeding programs.

## 2 Material and Methods

### 2.1 Plant Materials and Experimental Conditions

Six advanced tomato lines at the F6 generation, namely TAL101‐1, TAL101‐2, TAL102, TAL103, TAL104, and TAL105, were evaluated for their responses to high‐temperature stress under protected cultivation. The tomato breeding program at the University of Sulaimani developed the lines using parental germplasm originally obtained from the Vegetable Breeding Laboratory at Oregon State University. TAL101‐1 and TAL101‐2 were derived from the Indigo Rose × Santiam cross; TAL102, TAL103, and TAL104 from the Indigo Rose × Gold Nugget cross; and TAL105 from the Medford × Gold Nugget cross. Table 1 provides information on the pedigree, parental background, and relevant characteristics of the parental cultivars.

**TABLE 1:** Pedigree, parental background, and origin of the advanced tomato lines evaluated in this study.

Line	Generation	Source of parental germplasm	Pedigree	Parental genetic background
TAL101‐1	F6	Oregon State University/University of Sulaimani breeding program	Indigo Rose × Santiam	Anthocyanin‐rich × early/rapid‐maturing tomato
TAL101‐2	F6	Oregon State University/University of Sulaimani breeding program	Indigo Rose × Santiam	Anthocyanin‐rich × early/rapid‐maturing tomato
TAL102	F6	Oregon State University/University of Sulaimani breeding program	Indigo Rose × Gold Nugget	Anthocyanin‐rich × compact, determinate, early yellow cherry tomato
TAL103	F6	Oregon State University/University of Sulaimani breeding program	Indigo Rose × Gold Nugget	Anthocyanin‐rich × compact, determinate, early yellow cherry tomato
TAL104	F6	Oregon State University/University of Sulaimani breeding program	Indigo Rose × Gold Nugget	Anthocyanin‐rich × compact, determinate, early yellow cherry tomato
TAL105	F6	Oregon State University/University of Sulaimani breeding program	Medford × Gold Nugget	Heat/dry‐condition‐adapted determinate slicing tomato × compact, early yellow cherry tomato

*Note:* The tomato breeding program at the University of Sulaimani developed all six advanced tomato lines using parental germplasm originally obtained from the Vegetable Breeding Laboratory at Oregon State University. The lines were evaluated at the F6 generation. TAL101‐1 and TAL101‐2 originated from the same Indigo Rose × Santiam cross, whereas TAL102, TAL103, and TAL104 originated from the Indigo Rose × Gold Nugget cross. TAL105 originated from the Medford × Gold Nugget cross. Descriptions of parental characteristics are based on the breeding information available for the parental cultivars used in the development of these lines.

Seeds were initially sown in 36‐well trays filled with sterilized peat moss (TS 1, Klasmann‐Deilmann GmbH) and maintained in a controlled glasshouse at 23/18°C ± 2°C day/night temperature, 65% ± 10% relative humidity, and a 14/10 h light/dark photoperiod. These conditions were used solely for seed germination and seedling establishment and did not constitute the heat‐stress treatment.

At the four‐ to five‐true‐leaf stage, seedlings were transplanted into a naturally ventilated plastic high tunnel in late April and remained there until the end of August. The high tunnel lacked a cooling system or automated temperature control. Under typical production practices in the Kurdistan Region, tomato seedlings are transplanted into plastic houses in mid‐ to late February to reduce exposure of flowering and early fruit development to severe summer temperatures. In contrast, the later transplanting date in the present study was deliberately chosen to expose plants to progressively increasing temperatures during both vegetative and reproductive development.

After transplanting, all six tomato lines were exposed to the same naturally occurring high‐temperature environment without artificial heating or cooling. As shown in Figure 1, temperatures inside the high tunnel rose steadily from approximately 18°C–26.5°C in April to 25°C–47.8°C in August. Maximum temperatures exceeded 38°C from May/June onward, reaching approximately 47.8°C in August. Thus, the experimental conditions represented prolonged exposure to naturally occurring high temperatures throughout plant development rather than a short‐term, artificially imposed heat‐shock treatment.

**FIGURE 1:**
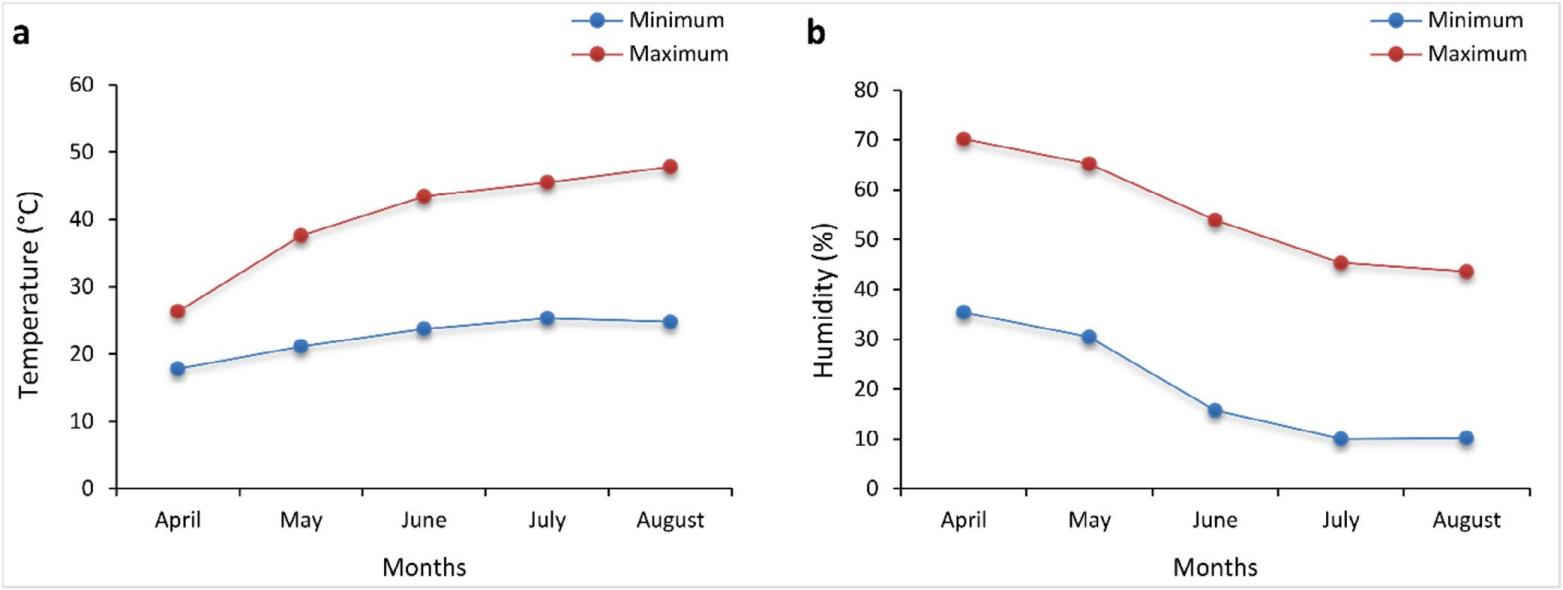
Temperature (a) and humidity (b) inside the plastic‐house throughout the study period.

### 2.2 DNA Isolation

Genomic DNA was isolated from freshly collected leaf tissue using the method reported by (Karim et al. 2025). Samples were stored at −20°C until analysis. The extracted DNA was run on a 1.3% agarose gel for visualization, and DNA concentration and purity were measured using a NanoDrop spectrophotometer (NanoPLUS‐MAANLAB AB, Sweden).

### 2.3 PCR Amplification of CDDP Markers

PCR amplification of CDDP markers (Table 2). Genetic diversity among the six advanced tomato lines was assessed using six Conserved DNA‐Derived Polymorphism (CDDP) primers, following (Rasul et al. 2022; Tahir, Lateef, et al. 2023). CDDP is a gene‐targeted molecular marker system based on conserved sequences of functionally characterized plant genes. The six primers used in this study were ABP1‐1, ERF1, ERF2, Knox1, WRKYR1, and WRKY‐R2, targeting conserved regions of the ABP1, ERF, KNOX, and WRKY gene families (Table 2). These markers were selected to provide gene‐targeted polymorphic profiles for assessing molecular diversity and genetic relationships among the advanced lines. The PCR reaction mixture contained 11 μL of master mix buffer (AddStart Taq Master, Addbio, Korea), 7 μL of extracted DNA (50 ng), 6 μL of primer (10 μM; Addbio, Korea), and 6 μL of deionized water, giving a final volume of 30 μL. PCR amplification was performed using a standard thermocycler (Prime, UK) with an initial denaturation at 94°C for 10 min, followed by 36 cycles of 94°C for 1 min, 50°C for 1 min, and 72°C for 2 min, with a final extension at 72°C for 10 min. Amplified products were separated by electrophoresis on 1.5% agarose gels prepared in 1× TBE buffer, stained with ethidium bromide (0.15 μg/mL), and electrophoresed at 90 V for 80 min. Gels were visualized under UV illumination and documented using a digital gel documentation system.

**TABLE 2:** CDDP primers, target gene families, primer sequences, and annealing temperature used for molecular characterization of the advanced tomato lines.

CDDP primer	Target gene/gene family	Primer sequence (5′–3′)	Annealing temperature (°C)
ABP1‐1	ABP1 (auxin‐binding protein 1)	ACSCCSATCCACCGC	50
ERF1	ERF (ethylene response factor)	CACTACCCCGGSCTSCG	50
ERF2	ERF (ethylene response factor)	GCSGAGATCCGSGACCC	50
Knox1	KNOX (knotted homeobox)	AAGGGSAAGCTSCCSAAG	50
WRKYR1	WRKY transcription factor	GTGGTTGTGTCTTGCC	50
WRKY‐R2	WRKY transcription factor	GCCCTCGTASGTSGT	50

### 2.4 Experimental Design

Prior to transplanting, the plastic‐house soil was plowed, leveled, and prepared for planting. The experiment was arranged in a randomized complete block design (RCBD) with three replications (blocks), with each block including all six advanced tomato lines. Each genotype was represented by six plants per replication. Plants were spaced 40 cm apart within rows, and 120 cm between blocks. The plants were arranged in a zigzag pattern to ensure uniform spacing and optimal use of the growing area. For data collection, three centrally located plants per genotype within each replication were selected, and their average values were used for all measured traits.

### 2.5 Experimental Site

The experiment was carried out during the growing season at the Horticulture Department research farm of the College of Agricultural Engineering Sciences, University of Sulaimani, Sulaymaniyah, Kurdistan Region, Iraq. The site is located at 35°32′9.6″N latitude and 45°21′54″E longitude, with an elevation of 741 m above sea level. The study was conducted under uncontrolled plastic‐house conditions to assess the tolerance of new advanced tomato lines to heat stress during the growing season. The plastic‐house was covered with a 200 μm‐thick transparent polyethylene film. Plastic‐house conditions were recorded throughout the experimental period (Figure 1).

### 2.6 Measurements

#### 2.6.1 Yield and Yield Components Traits

The following measurements were taken to assess yield and yield component traits. Plant height (PLH) was measured using a measuring tape from the soil surface to the highest tip of the plant. Three plants per replication were measured, and their average height was used as the representative value for that replication. The number of branches per plant (NBP) was determined by counting fully mature branches on three plants and averaging the counts. Flower diameter (FlD) was measured using a metric caliper, taking six randomly selected flowers per plant from three plants per replication; the diameter was recorded from edge to edge through the center of the flower, and the average was calculated. Cone length (CL) and style length (SL) were measured similarly with the caliper, but from base to top. The number of flowers per cluster (NFlC) was obtained by counting six clusters per plant for three plants and averaging. The number of clusters per branch (NClB) was measured by counting clusters on three branches per plant for three plants per replication and averaging. The number of clusters per plant (NClP) was then calculated by multiplying the number of branches per plant by the number of clusters per branch. The number of flowers per plant (NFlP) was computed as the product of clusters per plant and flowers per cluster. The number of fruits per cluster (NFC) was determined by counting fruits in six randomly selected clusters per plant, across three plants per replication, and averaging. The number of fruits per plant (NFP) was counted at the ripe stage when more than 80% of the fruits had matured, using three plants per replication and averaging. Fruit set percentage (FS) was calculated as the ratio of total fruit number per plant to total flower number per plant for three plants per replication, and the average was recorded. Fruit weight (FW) was measured by weighing ten fruits per plant, dividing by ten to obtain the single fruit weight for three plants per replication, and averaging. Leaf moisture percentage (LFM) and fruit moisture percentage (FM) were both determined by weighing fresh samples using a calibrated sensitive balance, then drying them in an oven at 72°C until a constant weight was achieved; the moisture percentage was calculated from the weight loss.

#### 2.6.2 Phytochemical Traits

Six fruits from each genotype were thawed at 4°C in a refrigerator, then homogenized for 1 min using an electronic blender to create a uniform sample. From each homogenate, 100 mL aliquots were taken for further analysis (Mohammed et al. 2024). All measurements were expressed on a fresh weight (FW) basis.

$$\mathrm{Dry}\;\text{matter}\%=\frac{\mathrm{Dry}\;\text{weight}}{\text{Fresh weight}\;}\times 100$$

Fruit moisture content (FMC%) was determined by first recording the fresh weight of each sample, then oven‐drying at 65°C until a constant dry weight was achieved. The moisture percentage was then calculated using the following equation:

$$\mathrm{FMC}\;\left(\%\right)=\frac{\text{Fresh weight}-\text{Oven}\;\mathrm{dry}\;\text{weight}}{\text{Fresh weight}}\times 100$$

Lipid peroxidation (LP) was assessed by measuring malondialdehyde (MDA) levels in leaf extracts of tomato plants, which indicate membrane oxidative damage caused by heat stress. The method followed Halshoy et al. (2025). Briefly, 30 μL of plant extract was placed into a test tube, and 2 mL of 0.5% (w/v) thiobarbituric acid prepared in 20% (w/v) trichloroacetic acid was added. The mixture was incubated in a water bath at 95°C for 30–31 min. Immediately after incubation, the reaction was stopped by cooling the samples in an ice bath, producing a pink coloration. The samples were then centrifuged at 4000 × g for 12 min. The supernatant's absorbance was measured at 532 and 600 nm using a UV–visible spectrophotometer. The absorbance at 532 nm reflects MDA content, while the reading at 600 nm was used to correct for nonspecific turbidity by subtraction. Lipid peroxidation was expressed as nmol/g fresh weight (FW) and calculated using the appropriate formula.

$$\mathrm{LP}=\frac{\mathrm{AB}532-\mathrm{AB}600\times 1000\times \mathrm{VL}}{\mathrm{EC}\times \text{WE}}$$

where AB_532_ = absorbance at 532 nm, AB_600_ = absorbance at 600 nm, VL = volume of extract (mL), WE = fresh weight of the sample (g), EC = extinction coefficient (155 mM/cm).

Proline content (PC) in tomato leaf extracts was determined according to the method described by Tahir, Rasul, and Lateef (2023). Briefly, 2 mL of leaf extract was mixed with 2 mL of acid ninhydrin reagent, prepared by dissolving 2.5 g of ninhydrin in 60 mL of glacial acetic acid and 40 mL of 6 M phosphoric acid, along with 2 mL of glacial acetic acid. The reaction mixture was incubated at 100°C for 60 min in a water bath. After incubation, the samples were cooled to room temperature, and 4 mL of toluene was added, followed by thorough mixing. The organic layer containing the chromophore was separated, and its absorbance at 520 nm was measured against a toluene blank using a UV–visible spectrophotometer. A standard proline solution was prepared by dissolving 10 mg of proline in 10 mL of deionized water to create a concentration of 1 mg/mL. This stock solution was serially diluted to produce standard concentrations ranging from 0.1 to 1 mg/mL for constructing a calibration curve. Proline content was expressed as micrograms of proline per gram of fresh weight (μg proline/g FW) and calculated based on the standard curve equation.

$$\mathrm{PC}=\frac{V}{W}\times C$$

where V is the total extract volume (mL), W is the fresh weight of the plant sample (g), and C is the proline concentration derived from the standard curve.

The soluble sugar content (SSC) of leaf and fruit extracts was measured following the method of Tahir et al. (2024). Briefly, 0.3 mL of the sample extract or glucose standard solution was mixed with 2.7 mL of anthrone reagent, prepared by dissolving 0.15 g of anthrone in a mixture of 84 mL sulfuric acid and 16 mL deionized water. The reaction mixture was heated in a boiling water bath for 5 min, then cooled to room temperature, and the absorbance was measured at 620 nm using a UV–visible spectrophotometer. A glucose standard solution was prepared by dissolving 10 mg of glucose in 10 mL of deionized water to create a concentration of 1 mg/mL. This stock was further diluted to prepare a series of standards ranging from 0.1 to 1 mg/mL to construct a calibration curve. The SSC was expressed as micrograms of glucose per gram of fresh weight (μg glucose/g FW) and calculated using the standard curve equation.

$$\mathrm{SSC}=\frac{V}{W}\times C$$

where V is the volume of the extract (mL), W is the fresh weight of the plant sample (g), and C is the glucose concentration determined from the standard curve.

The total phenolic content (TPC) of tomato leaf and fruit extracts was measured using the Folin–Ciocalteu method as outlined by Halshoy et al. (2023); Mohammed et al. (2026). Briefly, 100 μL of plant extract was combined with 4 mL of Folin–Ciocalteu reagent and incubated at room temperature for 5 min. Next, 2 mL of 20% (w/v) Na_2_CO_3_ solution was added, and the mixture was kept in the dark at 38°C for 50 min. A blank was prepared by replacing the extract with distilled water. Gallic acid served as the standard, and a calibration curve was created with concentrations between 0.1 and 1 mg/mL. Absorbance was read at 765 nm using a UV–visible spectrophotometer, and TPC was expressed as μg gallic acid equivalents per gram of fresh weight (μg GAE/g FW).

The total flavonoid content (TFC) was determined using the aluminum chloride (AlCl_3_) colorimetric method described by Abdalrahman et al. (2026); Karim et al. (2026). Briefly, 0.5 mL of plant extract was mixed with 1.5 mL of 80% methanol, 0.1 mL of 10% (w/v) AlCl_3_, 0.1 mL of 1 M potassium acetate, and 2.8 mL of distilled water. The mixture was incubated at room temperature for 45 min. A blank was prepared by replacing the extract with distilled water. Quercetin served as the standard, with concentrations ranging from 0.1 to 1 mg/mL used to construct a calibration curve. The absorbance was measured at 415 nm, and TFC was expressed as μg of quercetin equivalents per gram of fresh weight (μg QE/g FW).

Total antioxidant activity (TAA) was measured using the DPPH (1‐diphenyl‐2‐picrylhydrazyl) radical scavenging method, following Karim et al. (2025); Tofiq et al. (2024). Briefly, 30 μL of plant extract was added to 1.7 mL of a methanolic DPPH solution. The mixture was thoroughly mixed and incubated in the dark at room temperature for 30 min. Absorbance was then measured at 517 nm with a UV–visible spectrophotometer to determine the antioxidant capacity of the samples.

### 2.7 Statistical Analysis

The experiment was conducted using a randomized complete block design (RCBD) with three replicates. Differences among cultivars for the measured traits were evaluated using analysis of variance (ANOVA). Mean comparisons were performed using the least significant difference (LSD) test at the *p* < 0.05 level. In addition, multivariate analyses, including correlation and principal component analyses (PCAs), were conducted.

Genetic variability parameters, including the environmental coefficient of variation (ECV), genotypic coefficient of variation (GCV), phenotypic coefficient of variation (PCV), genetic advance as a percentage of the mean (GAPM), and broad‐sense heritability (*H* ^2^), were estimated from variance components. All statistical analyses were conducted in R (version 4.5.1; R Core Team, 2025) using the packages agricolae, metan, ggplot2, ggrepel, factoextra, FactoMineR, and variability (Visalakshi et al. 2024).

For molecular data analysis, scorable bands were manually scored by assigning “1” for the presence and “0” for the absence of bands. The dendrogram and radar plot were generated using SRPLOT, following Tang et al. (2023). The polymorphism information content (PIC) values were calculated using the iMEC: Online Marker Efficiency Calculator, as described by Amiryousefi et al. (2018).

## 3 Results

### 3.1 Analysis of Variance and Least Significant Differences

The analysis of variance (ANOVA) revealed highly significant genotypic differences across almost all measured morphological, yield, and phytochemical traits, indicating substantial genetic variation among the six tomato advanced lines in their responses to heat stress (Tables 3 and 4). This variation reflects distinct adaptive mechanisms influencing both vegetative growth and metabolic adjustments under elevated temperatures.

**TABLE 3:** The mean squares from the analysis of variance of tomato advanced lines' yield and yield component traits grown under heat stress conditions, with significance levels indicated.

Variables	DF	PLH (cm)	NBP	FlD (mm)	CL (mm)	SL (mm)	NFlC	NClB	NClP	NFlP	NFC	NFP	FS (%)	FW (g)	LFM (%)	FM (%)
Genotypes	5	2108.1***	7.96***	24.51*	3.66***	4.61	10.27***	74.72***	4692***	375028***	2.92*	3566***	96.88*	91.26	19.03***	278.6***
Replications	2	328.2	1.56	0.54	0.01	2.09	1.50	1.06	178	37,802	0.34	942*	41.91	15.37	0.00	0.00
Residuals	10	110.7	0.77	5.32	0.24	1.45	0.77	3.99	173	19,435	0.82	225	21.66	37.60	0.00	0.00

*Note:* Significant codes: 0 ‘***’ 0.01 ‘*’ 0.05.

Abbreviations: CL, cone length; FlD, flower diameter; FM, fruit moisture percentage; FS, fruits set percentages; FW, fruit weight; LFM, leaf moisture percentage; NBP, number of branches/plant; NClB, number of clusters/branch; NClP, number of clusters/plants; NFC, number of fruits/cluster; NFlC, number of flowers/cluster; NFlP, number of flowers/plant; NFP, number of fruits/plant; PLH, Plant height; SL, style length.

**TABLE 4:** Mean squares from the analysis of variance of phytochemical traits in tomato advanced lines grown under heat stress, with significance‐level marks.

Variables	DF	LFLP (nmol/g FW)	LFPC (μg/g FW)	LFSSC (μg/g FW)	FSSC (μg/g FW)	LFTPC (μg/g FW)	FTPC (μg/g FW)	LFTFC (μg/g FW)	FTFC (μg/g FW)	LFDPPH (μg/g FW)	FDPPH (μg/g FW)
Genotypes	5	2.4671***	129970***	49762***	82220***	18654***	593.1***	2797.2***	93.19***	20561***	9613***
Replications	2	0.0002	25.00	3.00	338**	7.00	0.80	4.9	1.32*	82**	5.00
Residuals	10	0.0011	23.00	8.00	31.00	7.00	3.90	22.2	0.28	9.00	3.00

*Note:* Significant codes: 0 ‘***’ 0.001 ‘**’ 0.01 ‘*’ 0.05.

Abbreviations: FDPPH, antioxidant activity in fruit; FSSC, soluble sugar in fruit; FTFC, total flavonoids content in fruit; FTPC, total phenolic content in fruit; LFDPPH, antioxidant activity in leaf; LFLP, lipid peroxidation in leaf; LFPC, proline in leaf; LFSSC, soluble sugar in leaf; LFTFC, total flavonoids content in leaf; LFTPC, total phenolic content in leaf.

For the yield and yield components traits (Table 3), significant (*p* < 0.05 to *p* < 0.001) genotype effects were recorded for plant height (PLH), number of branches per plant (NBP), flower diameter (FlD), cone length (CL), number of flowers per cluster (NFlC), number of clusters per branch (NClB), number of clusters per plant (NClP), number of flowers per plant (NFlP), number of fruits per cluster (NFC), number of fruits per plant (NFP), fruit set percentage (FS), leaf moisture (LFM), and fruit moisture (FM). These differences confirm that the studied lines responded differentially to heat stress in terms of reproductive potential and structural development.

Among the lines, TAL105 consistently demonstrated superior vegetative and reproductive vigor, reaching a maximum plant height of 125.66 cm, the highest number of branches (7.33), and 15.00 clusters per branch, resulting in 110.33 clusters per plant and 1033 flowers per plant. This indicates that TAL105 maintained strong vegetative growth and reproductive capacity even under heat stress. Similarly, TAL101‐1 exhibited robust performance for height (118.33 cm) and clusters per plant (89.33), whereas TAL103 showed moderate expression across growth parameters but retained a high flower diameter (22.73 mm) and fruit moisture content (58.71%), which could enhance fruit quality under stress (Figure 3).

Contrastingly, TAL101‐2 recorded the lowest plant height (65 cm) and the fewest clusters per plant (25.68), but achieved the highest fruit set percentage (24.03%), suggesting a compensatory reproductive mechanism under high‐temperature conditions that prioritized fruit retention over vegetative growth. TAL102 and TAL104 also performed modestly for most growth traits but retained stable fruit weights (28.90 g and 17.06 g, respectively), implying partial tolerance. These significant yield differences highlight that heat stress imposed differential limitations on growth and reproduction among the lines, with TAL105 demonstrating the most effective adaptation, as evidenced by sustained biomass and reproductive output.

The phytochemical ANOVA (Table 4) also showed highly significant (*p* < 0.001) variation across genotypes for all biochemical indicators, underscoring substantial genotypic differences in oxidative stress response and protective metabolite accumulation. Lipid peroxidation (LFLP) varied sharply among lines, with TAL103 recording the highest value (4.36 nmol/g FW), indicating greater membrane damage and oxidative susceptibility. Conversely, TAL105 had one of the lowest peroxidation levels (2.62 nmol/g FW) alongside the highest proline accumulation (LFPC = 614.81 μg/g FW), reflecting enhanced osmotic adjustment and cellular protection.

Further, TAL105 and TAL104 exhibited elevated soluble sugar contents in fruit (FSSC≈1806–1801 μg/g FW) and total flavonoid contents (FTFC = 27.41 μg/g FW in TAL105), both of which contribute to Osmo protection and antioxidant buffering. TAL104 recorded the highest total phenolic content in leaves (395.78 μg/g FW) and remarkable antioxidant activities (LFDPPH = 1292.16 μg/g FW; FDPPH = 397.75 μg/g FW), suggesting a strong oxidative defense system. By contrast, TAL103, despite moderate sugar levels, had low proline (26.31 μg/g FW) and high levels of peroxidation, indicating greater heat sensitivity (Figure 3).

The LSD comparisons (Tables 5 and 6) further clarified these genotypic contrasts. For yield‐related traits, TAL105 significantly surpassed all other lines in plant height, branching, cluster formation, flower and fruit numbers per plant, and fruit weight. This combination of morphological robustness and heat‐stable yield confirms TAL105's overall tolerance and adaptability. TAL101‐1 followed closely in general performance, while TAL101‐2 maintained effective reproductive compensation through higher fruit set. On the biochemical side, TAL105 combined the highest proline and flavonoid contents with strong antioxidant activity, reflecting active physiological defense against heat‐induced oxidative stress. TAL104, though weaker in vegetative growth, excelled in phenolic and antioxidant indices, demonstrating an alternative biochemical adaptation pathway. Meanwhile, TAL103's high lipid peroxidation signified oxidative vulnerability, aligning with its relatively reduced yield.

**TABLE 5:** The proportion of various variables, including Environmental Coefficient of Variance (ECV), Genotypic Coefficient of Variance (GCV), Phenotypic Coefficient of Variance (PCV), Genetic Advance as a percentage of the mean (GAPM), and Broad Sense Heritability (*H* ^2^), of the yield and yield component traits of advanced tomato lines grown under heat stress conditions.

Variability	PLH	NBP	FlD	CL	SL	NFlC	NClB	NClP	NFlP	NFC	NFP	FS	FW	LFM	FM
ECV (%)	11.67	19.08	10.50	7.37	15.28	11.93	19.43	25.21	34.31	26.91	33.16	36.71	27.87	0.00	0.00
GCV (%)	28.63	34.01	11.52	16.18	13.03	24.26	47.24	74.39	84.74	24.90	73.79	39.50	19.23	2.94	20.10
PCV (%)	30.92	38.99	15.59	17.78	20.08	27.04	51.08	78.55	91.42	36.66	80.90	53.93	33.86	2.94	20.10
GAPM (%)	54.62	61.09	17.54	30.34	17.43	44.85	90.00	145.15	161.80	34.84	138.64	59.60	22.49	6.06	41.42
H^2^	0.85	0.76	0.54	0.82	0.42	0.80	0.85	0.89	0.85	0.46	0.83	0.53	0.32	1.00	1.00

Abbreviations: CL, cone length; FlD, flower diameter; FM, fruit moisture percentage; FS, fruits set percentages; FW, fruit weight; LFM, leaf moisture percentage; NBP, number of branches/plant; NClB, number of clusters/branch; NClP, number of clusters/plants; NFC, number of fruits/cluster; NFlC, number of flowers/cluster; NFlP, number of flowers/plant; NFP, number of fruits/plant; PLH, plant height; SL, style length.

**TABLE 6:** The proportion of different variables, Environmental Coefficient of Variance (ECV), Genotypic Coefficient of Variance (GCV), Phenotypic Coefficient of Variance (PCV), Genetic Advance as percentage of mean (GAPM), and Broad Sense Heritability (*H* ^2^), of the phytochemical traits of the tomato advanced lines grown under heat stress conditions.

Variability	LFLP	LFPC	LFSSC	FSSC	LFTPC	FTPC	LFTFC	FTFC	LFDPPH	FDPPH
ECV (%)	1.03	2.17	0.29	0.33	0.95	4.19	3.47	3.16	0.24	0.46
GCV (%)	28.31	95.31	13.40	9.75	28.35	29.84	22.43	33.40	6.80	15.77
PCV (%)	28.33	95.33	13.40	9.75	28.37	30.14	22.69	33.55	6.80	15.78
GAPM (%)	58.29	196.28	27.60	20.07	58.38	60.88	45.66	68.50	14.00	32.48
*H* ^2^	0.99	0.99	0.99	0.99	0.99	0.98	0.97	0.99	0.99	0.99

Abbreviations: FDPPH, antioxidant activity in fruit; FSSC, soluble sugar in fruit; FTFC, total flavonoid content in fruit; FTPC, total phenolic content in fruit; LFDPPH, antioxidant activity in leaf; LFLP, lipid peroxidation in leaf; LFPC, proline in leaf; LFSSC, soluble sugar in leaf; LFTFC, total flavonoid content in leaf; LFTPC, total phenolic content in leaf.

The significant ANOVA effects and LSD separations (Tables 3 and 4; Figures 2 and 3) demonstrate that the six tomato advanced lines possess distinct strategies in coping with heat stress. TAL105 integrates vigorous growth, reproductive stability, and enhanced metabolic protection, representing a physiologically resilient genotype. TAL104, though less productive, maintains superior antioxidant potential, indicating a defense‐based adaptation. TAL101‐2, with its high fruit set despite small plant stature, exemplifies reproductive efficiency under stress. These genotypic patterns confirm that both morphological resilience and biochemical protection are central to tomato heat‐stress tolerance, and they highlight TAL105 and TAL104 as the most promising candidates for advancing breeding programs targeting thermotolerant tomato varieties.

**FIGURE 2:**
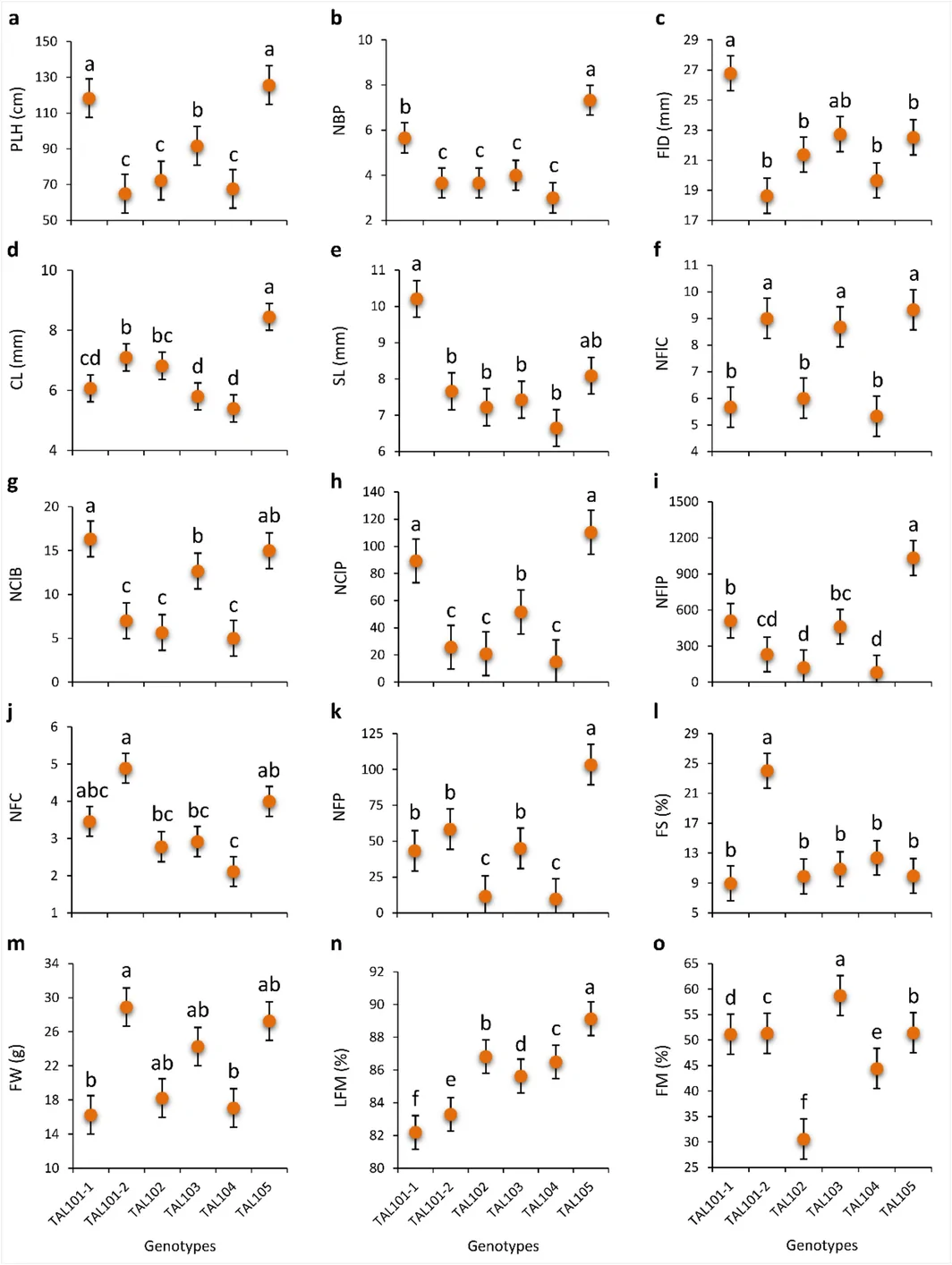
The least significant difference in comparing the means of the yield and yield components traits of the tomato advanced lines grown under heat stress conditions. (a) Plant height (PLH), (b) number of branches/plant (NBP), (c) flower diameter (FlD), (d) cone length (CL), (e) style length (SL), (f) number of flowers/cluster (NFlC), (g) number of clusters/branch (NClB), (g) number of clusters/plants (NClP), (i) number of flowers/plant (NFlP), (j) number of fruits/cluster (NFC), (k) number of fruits/plant (NFP), (l) fruits set percentages (FS), (m) fruit weight (FW), (n) leaf moisture percentage (LFM), and (o) fruit moisture percentage (FM). Treatments with the same letter are not significantly different.

**FIGURE 3:**
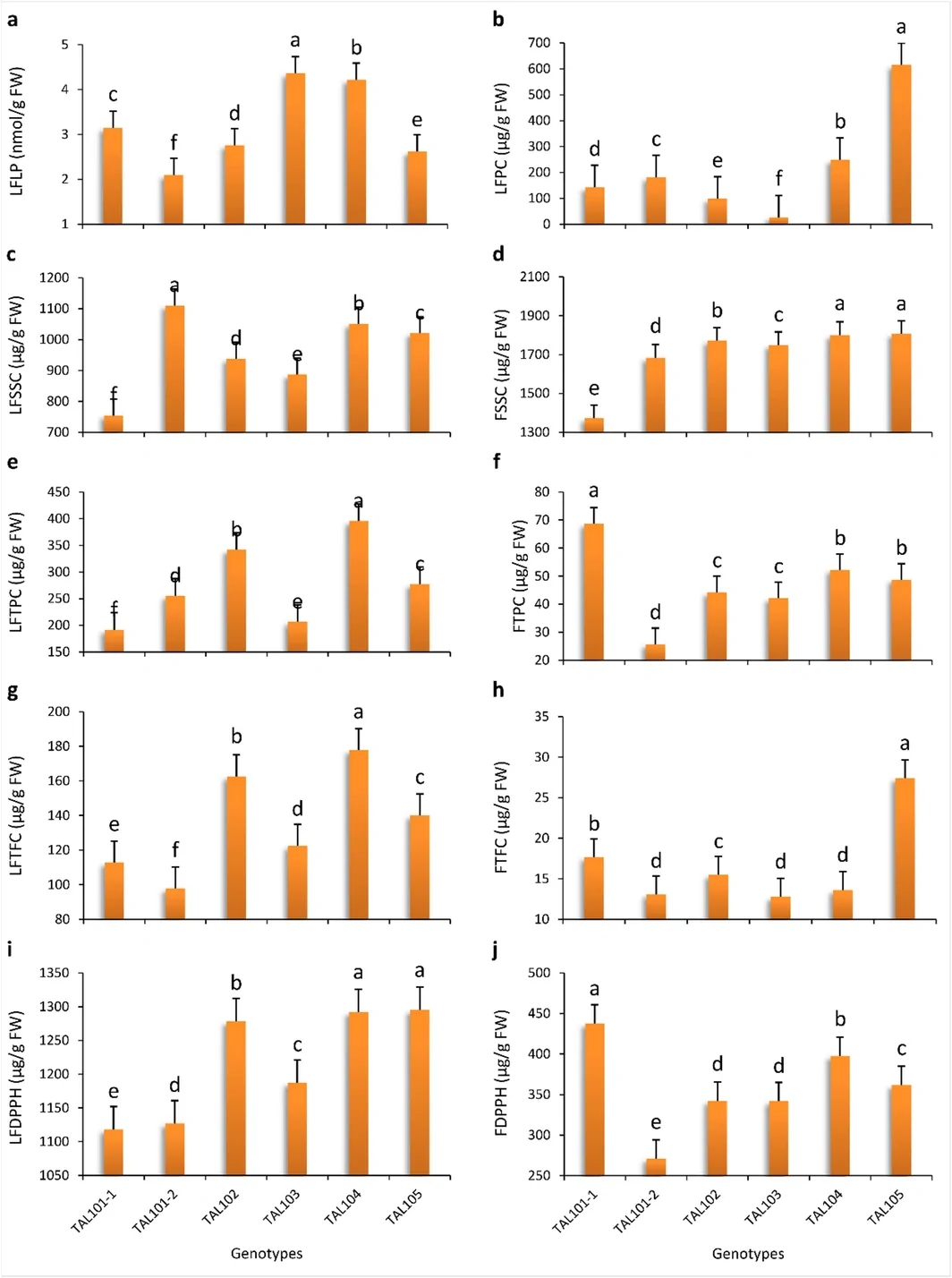
The least significant difference in comparing the means of the phytochemical traits of the tomato advanced lines grown under heat stress conditions. (a) lipid peroxidation in leaf (LFLP), (b) proline in leaf (LFPC), (c) soluble sugar in leaf (LFSSC), (d) soluble sugar in fruit (FSSC), (e) total phenolic content in leaf (LFTPC), (f) total phenolic content in fruit (FTPC), (g) total flavonoids content in leaf (LFTFC), (h) total flavonoids content in fruit (FTFC), (i) antioxidant activity in leaf (LFDPPH), and (j) antioxidant activity in fruit (FDPPH). Treatments with the same letter are not significantly different.

### 3.2 Proportion of Variability

The estimates of genetic parameters, including environmental (ECV), genotypic (GCV), and phenotypic coefficients of variation (PCV), as well as broad‐sense heritability (*H* ^2^) and genetic advance as a percentage of the mean (GAPM), are presented in Tables 5 and 6. These parameters provide crucial insight into the extent of genetic control, environmental influence, and selection potential of the studied traits under heat stress.

The magnitude of variability among genotypes was generally high for most yield‐related traits, indicating substantial genetic diversity and potential for selection even under stressful environmental conditions.

The phenotypic coefficients of variation (PCV) were consistently higher than the genotypic coefficients of variation (GCV) across all traits, indicating some environmental influence on trait expression under heat stress. However, the relatively small differences between PCV and GCV across most traits indicate that environmental effects were moderate and that a strong genetic component governs trait variability.

Among morphological and yield components, the number of flowers per plant (NFlP) and number of fruits per plant (NFP) exhibited exceptionally high GCV values of 84.74% and 73.79%, respectively, accompanied by similarly high PCV values (91.42% and 80.90%). This suggests that these reproductive traits are highly variable and largely genetically controlled, making them effective selection indices for heat tolerance. Similarly, the number of clusters per plant (NClP) showed a GCV of 74.39% and a PCV of 78.55%, indicating robust genetic control of cluster production even under elevated temperature conditions.

Plant height (PLH) and number of branches per plant (NBP) showed high heritability values of 0.85 and 0.76, respectively, indicating that these structural traits contributed substantially to the observed phenotypic variation under the evaluated high‐temperature conditions. Traits such as cone length (CL) and number of clusters per branch (NClB) also exhibited high heritability (0.82 and 0.85), indicating reliable transmission to the next generation. In contrast, style length (SL) and fruit weight (FW) showed moderate heritability (0.42 and 0.53), suggesting that they are more influenced by environmental variation and may respond less predictably to selection.

The genetic advance as a percentage of mean (GAPM) further emphasized the traits' selection potential. Exceptionally high GAPM values were observed for the number of clusters per plant (145.15%), the number of fruits per plant (138.64%), and the number of flowers per plant (161.80%), indicating that substantial improvement can be achieved through selection even under heat‐stress conditions. These traits, therefore, represent key targets for breeding programs aiming to enhance yield potential in heat‐tolerant genotypes. Moderate GAPM values for plant height (54.62%), branch number (61.09%), and cone length (30.34%) also point to considerable genetic gain under continued selection.

Collectively, these findings suggest that traits related to reproductive output (clusters, flowers, and fruits per plant) are under strong genetic control, with high heritability and variability. This genetic consistency under heat stress underscores their importance as reliable selection criteria for developing high‐yielding, heat‐tolerant tomato lines. The results reinforce earlier ANOVA and LSD findings that TAL105 and TAL101‐1 exhibit superior, genetically stable expression of these traits, making them prime candidates for advancement in heat‐stress breeding.

The estimates of variability and heritability for phytochemical traits demonstrated extremely high genotypic control, with nearly all traits exhibiting broad‐sense heritability values above 0.97, signifying minimal environmental influence on their expression under heat stress. This high heritability reflects the strong genetic regulation of biochemical stress responses, including osmolyte accumulation, antioxidant production, and membrane stability.

Proline accumulation in leaves (LFPC) displayed one of the highest heritability values (*H* ^2^ = 0.99) and an extraordinary genetic advance as a percentage of the mean (GAPM = 196.28%). This indicates that proline content is a highly heritable and strongly selectable trait, making it an excellent biochemical marker for heat tolerance. Similarly, lipid peroxidation (LFLP), which represents oxidative damage to membranes, showed high GCV (28.31%) and *H* ^2^ (0.99), suggesting that genetic differences significantly influence stress‐induced lipid degradation among genotypes.

Other osmotic and antioxidant traits, such as soluble sugars in leaf (LFSSC) and fruit (FSSC), exhibited GCV and PCV values of 13.40% and 9.75%, respectively, with very high heritability (0.99), indicating a substantial genetic contribution to variation in soluble sugar content under the evaluated high‐temperature conditions. Total phenolic content in leaf (LFTPC) and fruit (FTPC) also exhibited heritability values exceeding 0.98, with GAPM values of 58.38% and 60.88%, respectively, indicating consistent inheritance of antioxidant‐related compounds across genotypes.

Likewise, flavonoid contents (LFTFC, FTFC) and antioxidant activities (LFDPPH, FDPPH) showed uniformly high heritability (0.97–0.99) and moderate‐to‐high genetic gains (45.66%–68.50% for flavonoids and 14.00%–32.48% for antioxidant activities). These findings confirm that secondary metabolite accumulation is under tight genetic control and largely unaffected by environmental fluctuations. The high heritability estimates and associated genetic gains indicate considerable potential to use these traits as selection criteria in breeding programs aimed at improving performance under high‐temperature conditions.

The combined variability and heritability analyses (Tables 5 and 6) highlight that both yield‐related and phytochemical traits exhibit high genetic determination under heat stress, although the latter showed higher heritability estimates, indicating a greater genetic contribution to their observed phenotypic variation under the experimental conditions. Yield components, such as the number of clusters and fruits per plant, exhibit high heritability and genetic advance, suggesting they are reliable indicators of heat tolerance associated with reproductive efficiency. Meanwhile, biochemical traits such as proline, soluble sugars, and phenolic and flavonoid compounds exhibit nearly complete genetic determination, making them robust physiological markers of tolerance.

These results emphasize that the studied tomato lines harbor diverse yet heritable adaptive mechanisms, morphological, reproductive, and biochemical, that can be exploited through selection. The combination of high heritability and high genetic advance in both yield and phytochemical parameters underscores the effectiveness of selection‐based breeding for improving heat tolerance. Notably, TAL105's superior performance across most heritable yield and biochemical traits reinforces its potential as a leading thermotolerant genotype, while TAL104's high levels of phenolic and antioxidant expression highlight its suitability for breeding programs targeting heat resilience in biochemical traits.

### 3.3 Multivariate Correlation

The Pearson correlation matrix (Figure 4) revealed a complex, highly structured network of significant associations among morphological, reproductive, and phytochemical traits across the six advanced tomato lines evaluated under heat stress conditions. These interrelationships highlight the integrative nature of thermotolerance, where plant architecture, reproductive efficiency, and biochemical defense mechanisms operate in a coordinated manner.

**FIGURE 4:**
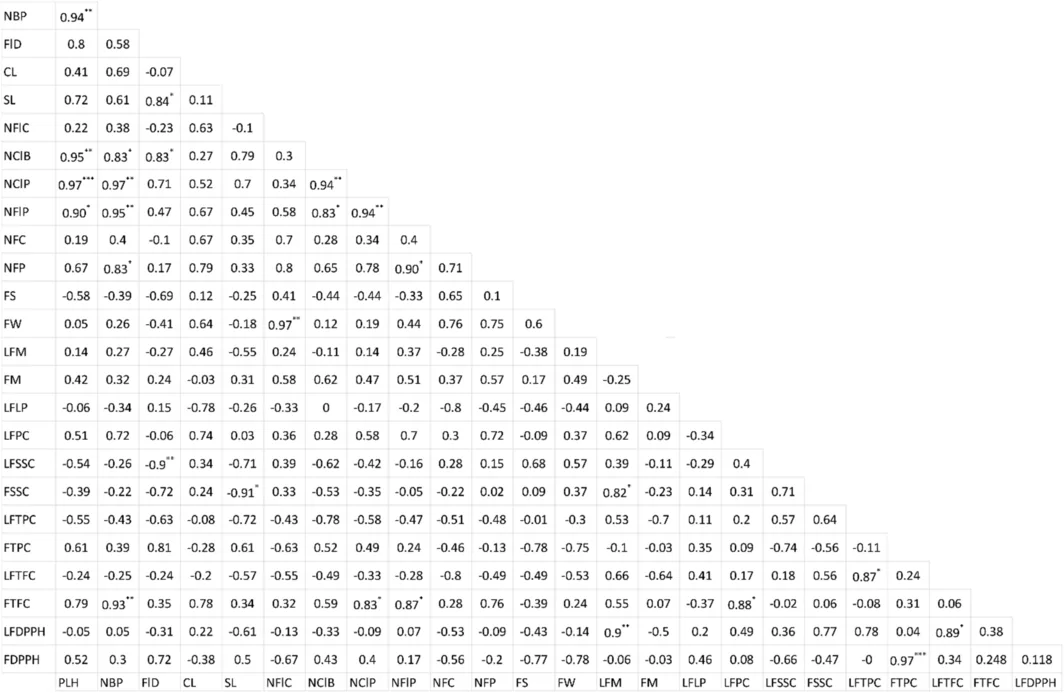
Multivariate correlation of the yield and yield components and phytochemical traits of the tomato advanced lines grown under heat stress conditions. Plant height (PLH), number of branches/plant (NBP), flower diameter (FlD), cone length (CL), style length (SL), number of flowers/cluster (NFlC), number of clusters/branch (NClB), number of clusters/plants (NClP), number of flowers/plant (NFlP), number of fruits/cluster (NFC), number of fruits/plant (NFP), fruits set percentages (FS), fruit weight (FW), leaf moisture percentage (LFM), fruit moisture percentage (FM), Lipid Peroxidation in Leaf (LFLP), Proline in Leaf (LFPC), Soluble Sugar in Leaf (LFSSC), Soluble Sugar in Fruit (FSSC), Total Phenolic Content in Leaf (LFTPC), Total Phenolic Content in Fruit (FTPC), Total Flavonoids Content in Leaf (LFTFC), Total Flavonoids Content in Fruit (FTFC), Antioxidant Activity in Leaf (LFDPPH), and Antioxidant Activity in Fruit (FDPPH). Significant codes: 0 ‘***’ 0.001 ‘**’ 0.01 ‘*’ 0.05. (−) refers to the negative correlation.

Strong, highly significant positive correlations were observed among most morphological and yield‐related traits, indicating that maintaining vegetative vigor under heat stress is closely associated with enhanced reproductive performance. PLH exhibited very strong positive correlations with NBP (*r* = 0.94), NClP (*r* = 0.97), and NFlP (*r* = 0.90). These relationships suggest that structurally robust plants have a greater capacity to produce assimilates and support reproductive development under thermal stress. Similarly, the strong association between NBP and NClP (*r* = 0.97) indicates that increased branching directly contributes to cluster formation, thereby improving yield potential.

Reproductive traits were also tightly interconnected. NClP showed a strong positive correlation with NFP (*r*≈0.90), indicating that fruit production is largely determined by the plant's ability to sustain cluster development under heat stress. Likewise, NFlC was positively associated with NFC (*r*≈0.70), reflecting efficient flower‐to‐fruit conversion in heat‐tolerant genotypes.

FW demonstrated a very strong positive correlation with FlD (*r* = 0.97) and a strong association with NFC (*r* = 0.76), indicating a coordinated developmental relationship between floral organ size and final fruit biomass. This linkage underscores the importance of reproductive organ development in maintaining fruit quality under elevated temperatures.

In contrast, FS showed negative correlations with key vegetative traits, including PLH (*r* = −0.58) and NBP (*r* = −0.39), suggesting a trade‐off between vegetative growth and reproductive allocation under heat stress. This pattern indicates that excessive investment in vegetative structures may limit the availability of assimilates for fruit set. The behavior of TAL101‐2, which exhibited reduced vegetative growth but higher FS (24.03%), exemplifies an adaptive strategy based on reproductive efficiency.

The relationships between morphological and biochemical traits further clarify the mechanisms of stress adaptation. LFPC showed positive correlations with NBP (*r* = 0.72) and NFlP (*r* = 0.70), indicating that osmotic adjustment contributes to sustaining growth and flowering under heat stress.

Conversely, LFLP, an indicator of oxidative membrane damage, was negatively correlated with several yield components, including CL (*r* = −0.78), NFC (*r* = −0.80), and NFP (*r* = −0.45). These negative relationships demonstrate that oxidative stress directly impairs reproductive efficiency and yield stability. The relatively low LFLP levels observed in TAL105, coupled with its superior yield performance, further confirm that membrane stability is a key determinant of heat tolerance.

Soluble sugar content exhibited differential associations with growth and reproductive traits. LFSSC was negatively correlated with FlD (*r* = −0.90) and SL (*r* = −0.71), but positively associated with FS (*r* = 0.68), suggesting a regulatory role in reproductive resilience rather than vegetative development. In contrast, FSSC showed a strong positive correlation with LFM (*r* = 0.82), indicating that osmotic adjustment contributes to maintaining tissue hydration and fruit integrity under heat stress.

Biochemical traits displayed strong interrelationships, reflecting a coordinated antioxidant defense system. LFTPC was positively correlated with FTPC (*r* = 0.61), indicating partial synchronization of phenolic accumulation between plant organs. LFTFC and FTFC exhibited a very strong positive correlation (*r* = 0.87), while their associations with LFTPC were moderate (*r*≈0.64–0.66), suggesting coordinated but distinct regulatory pathways for these secondary metabolites.

Antioxidant activities (LFDPPH and FDPPH) were strongly associated with phenolic and flavonoid contents, particularly LFTFC (*r* = 0.89) and FTPC (*r* = 0.97), indicating that antioxidant capacity is largely driven by these compounds. Additionally, LFDPPH showed a strong positive correlation with LFM (*r* = 0.90), suggesting that enhanced antioxidant activity contributes to improved water status and reduced oxidative damage. In fruits, FDPPH was strongly correlated with FTPC (*r* = 0.97) and weakly to moderately associated with FTFC (*r* = 0.34), indicating that phenolics play a dominant role in determining antioxidant capacity in fruit tissues.

The correlation matrix highlights that heat tolerance in tomato is a multi‐trait phenomenon, involving tightly linked morphological, reproductive, and biochemical components. High‐yield performance under heat stress was positively associated with PLH, NBP, NClP, and NFlP, and inversely associated with oxidative markers such as LFLP. Meanwhile, biochemical protectants including LFPC, soluble sugars (LFSSC and FSSC), phenolics (LFTPC and FTPC), and flavonoids (LFTFC and FTFC) were strongly interconnected and positively associated with antioxidant activities (LFDPPH and FDPPH), forming a physiological shield that supports yield maintenance.

These correlations reinforce the conclusion that superior lines, such as TAL105 and TAL104, integrate structural vigor and metabolic protection under heat stress conditions. TAL105 achieves tolerance through strong vegetative and reproductive development combined with biochemical efficiency, whereas TAL104 relies more on antioxidant and phenolic pathways to mitigate oxidative damage. Overall, these findings underscore the complex but synergistic relationships among developmental, reproductive, and biochemical traits in determining thermotolerance and provide essential guidance for multi‐trait selection in breeding programs aimed at improving tomato adaptation to high‐temperature environments.

### 3.4 Principal Component Analysis

Principal Component Analysis (PCA) effectively summarized the multidimensional variation among the six advanced tomato lines evaluated under heat stress and identified the key traits that contributed to their differentiation (Figures 5 and 6). The first two principal components (PC1 and PC2) explained a substantial proportion of the total variance (67.5%), representing the major axes of variation associated with growth, yield, and biochemical responses.

**FIGURE 5:**
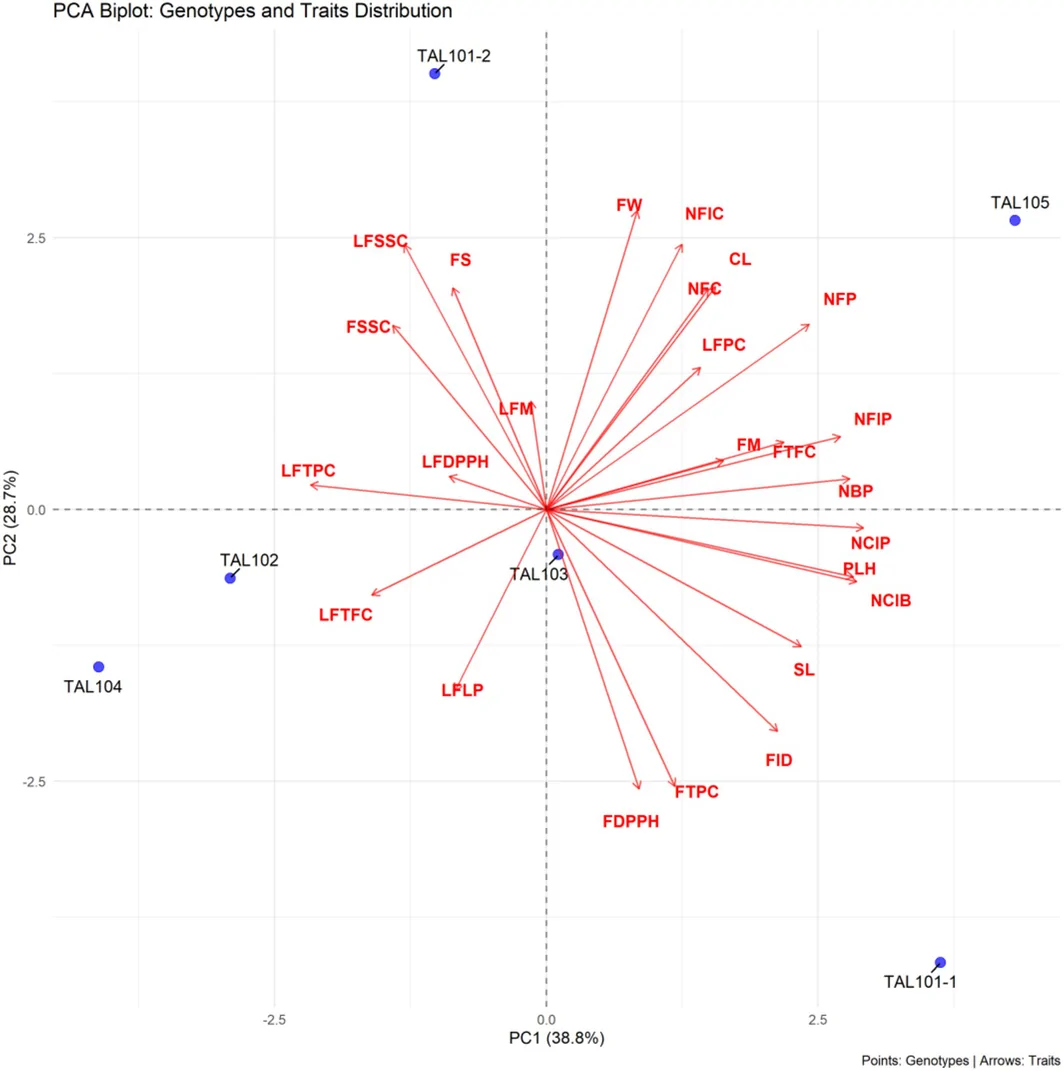
The PCA biplot showing the distribution of the tomato advanced lines according to the impact of the studied traits: Plant height (PLH), number of branches/plant (NBP), flower diameter (FlD), cone length (CL), style length (SL), number of flowers/cluster (NFlC), number of clusters/branch (NClB), number of clusters/plant (NClP), number of flowers/plant (NFlP), number of fruits/cluster (NFC), number of fruits/plant (NFP), fruit set percentages (FS), fruit weight (FW), leaf moisture percentage (LFM), fruit moisture percentage (FM), lipid peroxidation in leaf (LFLP), proline in leaf (LFPC), soluble sugar in leaf (LFSSC), soluble sugar in fruit (FSSC), total phenolic content in leaf (LFTPC), total phenolic content in fruit (FTPC), total flavonoids content in leaf (LFTFC), total flavonoids content in fruit (FTFC), antioxidant activity in leaf (LFDPPH), and antioxidant activity in fruit (FDPPH).

**FIGURE 6:**
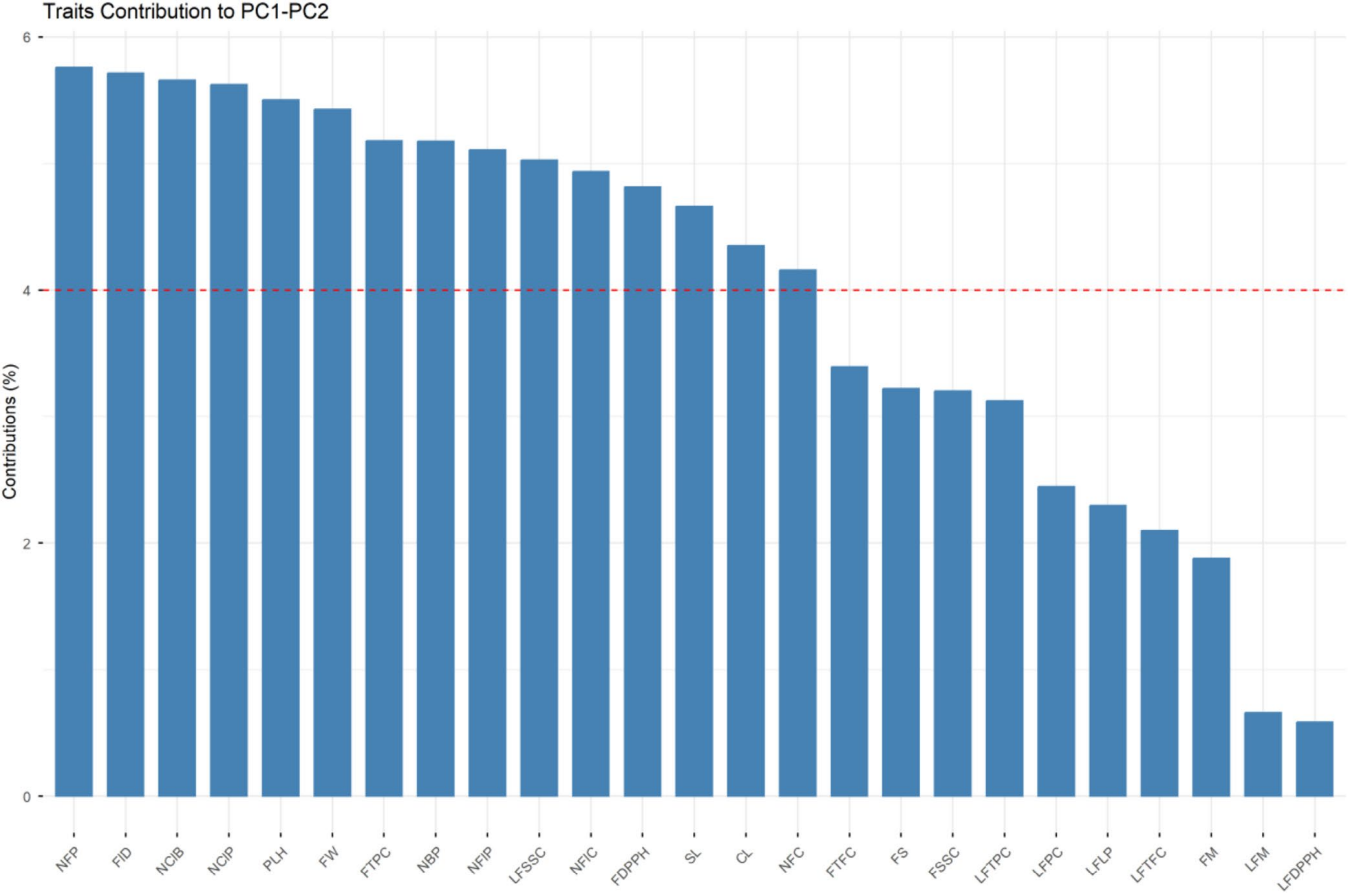
The trait contributions to PC1‐PC2: Plant height (PLH), number of branches/plant (NBP), flower diameter (FlD), cone length (CL), style length (SL), number of flowers/cluster (NFlC), number of clusters/branch (NClB), number of clusters/plants (NClP), number of flowers/plant (NFlP), number of fruits/cluster (NFC), number of fruits/plant (NFP), fruits set percentages (FS), fruit weight (FW), leaf moisture percentage (LFM), fruit moisture percentage (FM), Lipid Peroxidation in Leaf (LFLP), Proline in Leaf (LFPC), Soluble Sugar in Leaf (LFSSC), Soluble Sugar in Fruit (FSSC), Total Phenolic Content in Leaf (LFTPC), Total Phenolic Content in Fruit (FTPC), Total Flavonoids Content in Leaf (LFTFC), Total Flavonoids Content in Fruit (FTFC), Antioxidant Activity in Leaf (LFDPPH), and Antioxidant Activity in Fruit (FDPPH).

PC1 accounted for the largest proportion of variability and was primarily associated with morphological and yield‐related traits. High positive loadings were observed for PLH, NBP, FlD, NClB, NClP, NFlP, and FW, indicating that this component represents overall plant vigor and reproductive capacity. The strong contributions of these traits, all exceeding the average threshold (approximately 4%), confirm that genotypic variation along PC1 is largely driven by coordinated differences in structural growth and yield performance under heat stress. This pattern is consistent with the correlation analysis, where these traits exhibited strong positive interrelationships. Genotypes such as TAL105 and TAL101‐1 were positioned toward the positive side of PC1, reflecting their superior vegetative development, branching, cluster formation, and fruit production.

In contrast, PC2 was mainly defined by biochemical and physiological traits associated with stress response and metabolic protection. High positive loadings were observed for LFPC, LFTPC, FTPC, LFTFC, FTFC, LFDPPH, and FDPPH, indicating that these components represent antioxidant capacity and biochemical resilience. These traits were strongly intercorrelated in the correlation matrix, supporting their collective role in an integrated antioxidant defense system. High PC2 scores were primarily associated with TAL104, indicating its strong capacity to accumulate protective metabolites and activate antioxidant mechanisms. TAL105 also showed a positive but comparatively moderate association with PC2, reflecting its balanced integration of structural and biochemical adaptations.

LFLP exhibited a strong negative loading on PC2, indicating that this component also captures variation related to oxidative damage. Genotypes positioned on the negative side of PC2, particularly TAL103, were associated with higher lipid peroxidation and reduced antioxidant capacity, reflecting greater susceptibility to heat‐induced oxidative stress. This inverse relationship between LFLP and antioxidant traits is consistent with the correlation analysis, where oxidative damage was negatively associated with yield and biochemical protection.

The PCA biplot (Figure 6) illustrates the relationships among traits and genotypes within the reduced multivariate space. Three general groupings of genotypes were observed. The first group, comprising TAL105 and TAL101‐1, was positioned on the positive side of PC1 and moderately on PC2, indicating superior morphological performance combined with adequate biochemical stability. These genotypes were closely associated with key yield‐related traits such as NFP, NClP, and FW, as well as antioxidant activities (LFDPPH and FDPPH), suggesting their ability to maintain both productivity and oxidative protection under heat stress.

The second group, represented by TAL104, occupied the positive region of PC2 but was positioned closer to the origin on PC1. This placement reflects moderate yield performance but strong biochemical activity, driven by high phenolic and flavonoid accumulation. TAL104, therefore, represents a defense‐oriented adaptation strategy that relies primarily on metabolic protection rather than structural vigor.

The third group, including TAL101‐2, TAL102, and TAL103, was located toward the negative end of both components, indicating lower performance across morphological and biochemical traits. These genotypes were characterized by reduced growth, lower yield potential, higher LFLP, and weaker antioxidant activity. In particular, TAL103 showed a close association with LFLP, highlighting its susceptibility to oxidative damage and reduced thermotolerance.

The orientation of trait vectors in the PCA biplot further supports the relationships identified in the correlation matrix. Morphological and yield‐related traits such as PLH, NBP, NClB, NClP, and NFP were closely aligned, confirming their strong positive interdependence. In contrast, LFLP was oriented in the opposite direction to LFPC, phenolics, flavonoids, and antioxidant traits, illustrating a clear negative association between oxidative damage and biochemical protection. The close proximity of antioxidant activity vectors (LFDPPH and FDPPH) to phenolic and flavonoid traits further indicates that antioxidant capacity is strongly associated with the accumulation of these secondary metabolites.

Overall, the PCA revealed two primary and complementary adaptive axes underlying heat tolerance in tomato: a morphological–yield axis (PC1) and a biochemical–defense axis (PC2). Genotypes such as TAL105 demonstrated favorable positioning along both axes, combining strong vegetative growth and reproductive efficiency with effective biochemical protection, making it the most balanced and thermotolerant line. In contrast, TAL104 relied predominantly on biochemical mechanisms, while genotypes such as TAL103 exhibited limited integration of these adaptive responses and greater sensitivity to heat stress.

These findings are consistent with the ANOVA, heritability, and correlation analyses, confirming that heat tolerance in tomato is a complex, multidimensional trait that integrates morphological robustness and physiological resilience. Therefore, effective breeding strategies should simultaneously target both yield‐related and biochemical traits. Genotypes such as TAL105, which demonstrate stability across both dimensions, represent promising candidates for developing thermotolerant tomato cultivars with sustained productivity under high‐temperature environments.

### 3.5 Molecular Characterization

Molecular characterization of the six advanced tomato lines was conducted using stress‐related markers to assess genetic relationships and variation among genotypes under heat stress conditions. A hierarchical phylogenetic tree based on these markers is presented in Figure 7.

**FIGURE 7:**
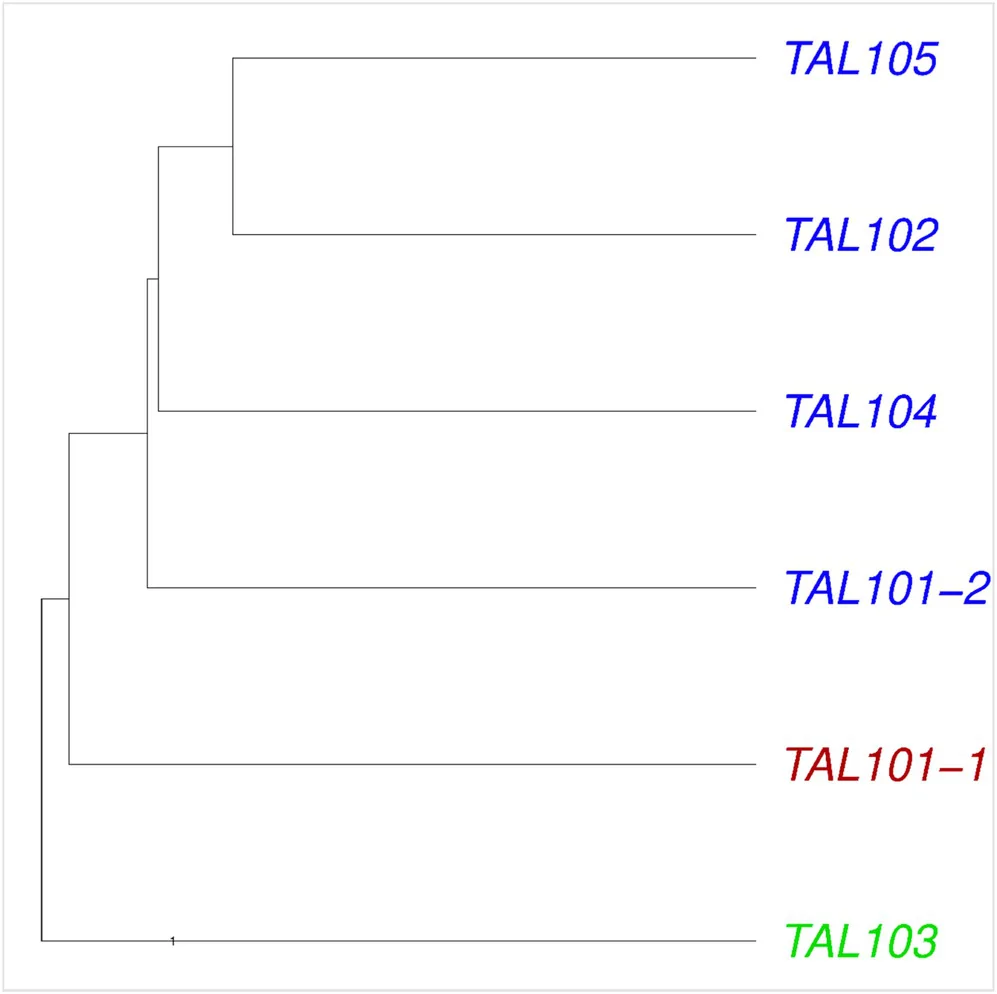
Phylogenetic tree illustrating the genetic relationships among six advanced tomato lines based on stress‐related molecular markers under heat stress conditions.

The phylogenetic analysis revealed clear genetic differentiation among the evaluated lines. The dendrogram grouped the genotypes into three main clusters. The first cluster included TAL105 and TAL102, indicating a close genetic relationship between them. The second cluster comprised TAL104 and TAL101‐2, reflecting a moderate level of genetic similarity. The third cluster consisted of TAL101‐1 and TAL103, with TAL103 forming the most distinct and distant branch in the tree, indicating greater genetic divergence than the other genotypes.

The distribution of genotypes across clusters corresponded with observable variation among lines in morphological, yield, and biochemical traits described in previous sections. Genotypes grouped in the phylogenetic tree generally exhibited comparable performance patterns under heat stress conditions, whereas genetically distant genotypes showed contrasting responses.

The profiles of stress‐related genes, including WRKYR1, WRKY‐R2, ERF1, ERF2, ABP1‐1, and Knox1, are illustrated in Figure 8 using radar plots. These figures summarize the relative expression or intensity of marker‐associated responses among the evaluated genotypes. The radar plots indicated variation in the relative marker profiles among the tomato lines (Figure 8a). Higher relative values for several markers were observed in some genotypes compared to others, while certain lines exhibited comparatively lower levels across multiple markers. In particular, variation was evident in markers associated with transcriptional regulation and stress response, including WRKY and ERF gene families.

**FIGURE 8:**
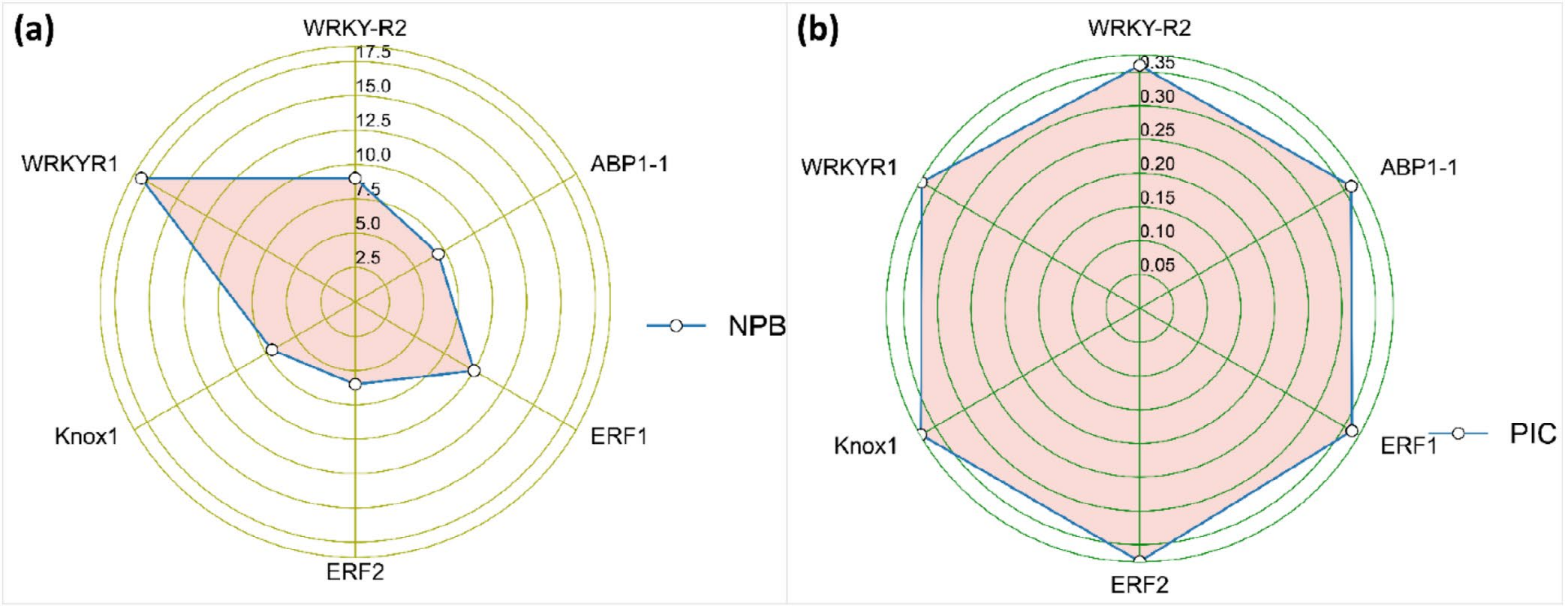
Radar plot showing the relative CDDP marker profiles generated using WRKY‐R1, WRKY‐R2, ERF1, ERF2, ABP1‐1, and Knox1 primers in six advanced tomato lines under heat‐stress conditions. (a) Number of polymorphic bands (NPB), (b) Polymorphism information content (PIC).

Differences in marker profiles were also observed among genotypes belonging to different phylogenetic clusters. Genotypes within the same cluster showed relatively similar marker activity patterns, whereas those in separate clusters displayed more distinct profiles. In contrast, TAL103 exhibited comparatively lower values for several markers and showed a distinct pattern relative to other genotypes. The molecular analysis revealed genetic diversity among the advanced tomato lines and variation in stress‐related marker profiles (Figure 8b). These results provide additional information on genetic differentiation and potential variability in stress‐response mechanisms among the evaluated genotypes.

## 4 Discussion

The response of tomato plants to heat stress involves a complex integration of morphological, physiological, and biochemical processes that collectively determine their tolerance and yield stability under high‐temperature conditions (Sousaraei et al. 2021; Visakh et al. 2025). Exposure to temperatures above the optimal range of 21°C–27°C during the day and 16°C–18°C at night (Hoshikawa et al. 2021) severely disrupts growth, pollination, and fruit set, thereby reducing productivity (Sulaiman and Sadiq 2020). Sustained temperatures exceeding 32°C–35°C can cause irreversible reproductive failure and oxidative damage (Ayenan et al. 2019; Sato et al. 2000). Within this physiological context, the present study revealed that the six tomato advanced lines exhibited significant genotypic variation across morphological, yield, and phytochemical traits, highlighting diverse adaptation strategies that could be exploited in breeding programs for thermotolerant cultivars (Fernández‐Crespo et al. 2022; Ruggieri et al. 2019).

### 4.1 Morphological and Yield Adaptations

Morphological and reproductive parameters were markedly affected by heat stress, consistent with prior reports that high temperatures inhibit vegetative development, reduce flower fertility, and lower fruit yield in tomato (Sulaiman and Sadiq 2020). The significant genotypic variation observed in traits such as plant height, number of branches, clusters, and flowers per plant, with heritability estimates exceeding 0.80 for most, indicates strong genetic control and selection potential (Bollier et al. 2023). Among the tested lines, TAL105 exhibited superior morphological and reproductive performance, maintaining the highest plant height (125.66 cm), branch number (7.33), clusters per plant (110.33), and flowers per plant (1033). This ability to sustain vigorous growth and reproductive development under heat aligns with earlier findings that structural vigor and sustained assimilate partitioning are critical for thermotolerance (Chaudhary et al. 2022; Pressman et al. 2002).

In contrast, TAL101‐2 maintained a relatively small vegetative structure but achieved the highest fruit‐set percentage (24.03%), representing a compensatory reproductive adaptation under thermal stress (Haque et al. 2021). Such resource allocation trade‐offs favoring reproductive efficiency over vegetative expansion have been reported as a successful survival strategy in heat‐stressed tomato (Sato et al. 2000; Xu et al. 2017). The high genotypic and phenotypic coefficients of variation (GCV and PCV) for yield‐related traits further demonstrate substantial additive genetic variance, confirming that selection based on these traits can accelerate breeding for heat tolerance (Zannat et al. 2023).

### 4.2 Biochemical and Physiological Mechanisms of Tolerance

At the biochemical level, the genotypes exhibited pronounced differences in their oxidative stress responses and osmoprotective capacity. TAL105 and TAL104 emerged as particularly resilient, exhibiting low lipid peroxidation (2.62 and 2.85 nmol/g FW, respectively) and elevated concentrations of protective metabolites. Low lipid peroxidation reflects reduced membrane damage, a primary marker of thermotolerance (Raja et al. 2020; Yang et al. 2019). Proline, which reached 614.81 μg/g FW in TAL105, functions as a key osmolyte stabilizing proteins and membranes, maintaining cellular hydration, and scavenging reactive oxygen species (ROS) under heat stress (Choudhury et al. 2017; Elkelish et al. 2020). Its high heritability (*H* ^2^ = 0.99) further underscores its reliability as a selection criterion (Rasheed et al. 2023).

TAL104 demonstrated exceptional antioxidant metabolism with the highest total phenolic content (395.78 μg/g FW) and antioxidant activity. This response reflects a defense‐oriented adaptation emphasizing ROS detoxification through phenolic and flavonoid biosynthesis (Fernández‐Crespo et al. 2022; Zhang et al. 2022). Enhanced accumulation of these secondary metabolites helps sustain oxidative balance during thermal stress (Ayenan et al. 2021; Jahan et al. 2022).

### 4.3 Interrelationships Among Traits and Adaptive Coordination

The correlation analyses provided further insight into the coordination of morphological, yield, and biochemical responses. Strong positive correlations among vegetative and reproductive traits (*r* > 0.90) indicate that vigorous vegetative growth directly supports reproductive potential and yield under stress (Ghabileh et al. 2024; Lee et al. 2022). The negative correlations between fruit set percentage and vegetative traits (e.g., *r* = −0.58 for plant height) suggest physiological trade‐offs in assimilate partitioning, in which certain genotypes channel more resources toward fruit retention when vegetative development is constrained (Sato et al. 2000).

Proline accumulation was positively correlated with growth and reproductive parameters (*r* = 0.70–0.72), demonstrating its critical role in sustaining flower and fruit formation under heat (Tonhati et al. 2020). Similarly, strong positive interrelationships among total phenolics, flavonoids, and antioxidant activities (*r* > 0.85) underscore the synergistic nature of secondary metabolite biosynthesis in enhancing oxidative defense (Choudhury et al. 2017). Negative correlations between lipid peroxidation and yield traits (*r* = −0.80) further highlight oxidative damage as a major limiting factor for fruit development (Camejo et al. 2006).

### 4.4 Multivariate Differentiation and Adaptive Strategies

The PCA results provided a comprehensive synthesis of these interactions. The first principal component (PC1) represented morphological and yield‐related attributes, while the second component (PC2) was dominated by biochemical and antioxidant parameters. This pattern delineates two major adaptation strategies: morphological–reproductive robustness and biochemical–defense resilience (Haakim et al. 2025; John and Stephen 2024). Genotypes such as TAL105 were strongly positioned along both PC1 and PC2, indicating superior integration of structural growth and biochemical protection. In contrast, TAL103 clustered negatively on both components, confirming its heat sensitivity. These patterns reaffirm that effective thermotolerance in tomato requires a dual mechanism combining physical vigor and metabolic resistance (Ayenan et al. 2019; Singh et al. 2022).

### 4.5 Breeding Implications

The high heritability and genetic advance observed suggest substantial potential for genetic improvement of heat tolerance through selection. Traits such as number of clusters, fruits per plant, proline, phenolic content, and antioxidant activity can serve as reliable selection indices (Raza et al. 2022; Siddiqui et al. 2015). Accordingly, TAL105 represents a model genotype combining high productivity with strong biochemical defenses. A combinatorial breeding approach incorporating both structural resilience and antioxidant efficiency is recommended to achieve durable resilience under global warming scenarios (Hafeez et al. 2023; Teresia et al. 2025). These findings confirm that thermotolerance is defined by the synergistic interaction among morphological robustness, reproductive efficiency, and metabolic defense mechanisms (Ayenan et al. 2019; Li et al. 2026).

### 4.6 Integrated Molecular Validation of Heat Tolerance Mechanisms

The incorporation of molecular characterization alongside morphological, yield, and biochemical analyses provides a more comprehensive understanding of heat tolerance in the evaluated tomato lines. While correlation and principal component analyses identified key trait associations and the main axes of variation, the molecular results provide complementary genetic evidence, reinforcing the multidimensional nature of thermotolerance observed in this study.

The correlation matrix and PCA consistently demonstrated that heat tolerance is structured along two principal dimensions: a morphological–yield axis (PC1) and a biochemical–defense axis (PC2). Traits such as PLH, NBP, NClP, NFlP, and FW were strongly and positively associated, forming the structural basis for yield stability under heat stress. In parallel, biochemical traits including LFPC, phenolics, flavonoids, and antioxidant activities were tightly interlinked and negatively associated with LFLP, indicating that the ability to mitigate oxidative damage is a critical determinant of plant performance. These patterns highlight that both growth maintenance and cellular protection are essential components of thermotolerance.

The molecular analysis substantiates these findings by demonstrating that phenotypic and biochemical variation among genotypes is underpinned by distinct genetic relationships and differences in stress‐related marker profiles. The phylogenetic clustering separated the advanced lines into groups that closely correspond to their observed performance. For example, TAL105, which showed strong positive associations with morphological and yield traits and favorable positioning along both PCA axes, clustered with TAL102, indicating a shared genetic background linked to productive performance. TAL104, characterized by strong biochemical activity and high contribution to PC2, formed a separate cluster, reflecting its reliance on metabolic and antioxidant defense mechanisms. In contrast, TAL103, which exhibited high LFLP, weak antioxidant capacity, and negative PCA positioning, was genetically distant, confirming its classification as a heat‐sensitive genotype. Intermediate lines such as TAL101‐1 and TAL101‐2 showed moderate clustering and performance, indicating partial integration of adaptive traits.

Variation in stress‐responsive markers further supports these genotype‐specific responses. Markers associated with transcriptional regulation and stress signaling, particularly within the WRKY and ERF families, were more active in genotypes with higher antioxidant capacity and greater accumulation of phenolics and flavanols, such as TAL104 and TAL105. This alignment indicates that the biochemical traits identified in correlation and PCA analyses are supported by coordinated gene‐associated responses. Conversely, TAL103 exhibited comparatively lower marker activity, consistent with its higher oxidative damage and reduced biochemical protection. These relationships confirm that insufficient activation of stress‐responsive pathways is associated with increased susceptibility to heat stress.

The integration of molecular results with multivariate analyses highlights the biological relevance of the two adaptive strategies identified in this study. Genotypes associated with PC1 rely primarily on morphological vigor and reproductive efficiency, whereas those associated with PC2 depend on biochemical and antioxidant defense mechanisms. Molecular markers effectively bridge these dimensions by reflecting the regulatory processes underlying both growth and stress response. Genotypes such as TAL105 demonstrate a balanced integration of these mechanisms, combining structural performance with effective molecular and biochemical responses, while TAL104 represents a defense‐oriented strategy driven by enhanced metabolic activity.

Overall, the strong agreement among molecular, biochemical, and multivariate analyses confirms that heat tolerance in tomato is a complex, integrated trait governed by coordinated interactions between plant structure, metabolism, and gene‐associated responses. The molecular findings not only validate the patterns observed at the phenotypic level but also reveal the genetic basis underlying these responses. This integration significantly strengthens the study and highlights the value of combining molecular tools with conventional trait analysis. From a breeding perspective, such an approach enables more precise identification of thermotolerant genotypes, particularly those like TAL105 that exhibit stability across multiple response levels, thereby supporting the development of resilient tomato cultivars in high‐temperature environments.

The present experiment was designed as a screening and characterization study under naturally occurring high‐temperature conditions and therefore did not include a parallel optimal‐temperature control treatment. Consequently, the results allow comparison among genotypes under heat‐stress conditions but do not permit direct estimation of the percentage reduction in individual traits relative to each genotype's performance under optimal temperature. Future experiments incorporating contrasting temperature regimes will be valuable for quantifying genotype × temperature interactions and identifying genotypes that combine high productivity under optimal conditions with superior heat‐stress resilience.

## 5 Conclusion

The present study demonstrated substantial genotypic variation among advanced tomato lines under heat stress, highlighting the integration of morphological, reproductive, biochemical, and molecular mechanisms in determining thermotolerance. Traits related to reproductive performance, particularly clusters, flowers, and fruits per plant, exhibited high variability, heritability, and genetic advance, indicating strong selection potential. Biochemical parameters, including proline, phenolics, flavonoids, and antioxidant activities, showed high broad‐sense heritability estimates, indicating a substantial genetic contribution to their phenotypic variation under the evaluated high‐temperature conditions. PCA demonstrates trait/genotype associations and patterns of variation, but it does not establish causation. Therefore, avoid implying that antioxidant defense caused higher yield unless supported by additional analysis. Molecular characterization using stress‐related markers further revealed clear genetic differentiation among the six tomato lines, with distinct clustering patterns and variation in the molecular profiles generated by WRKY, ERF, ABP1‐1, and Knox1 CDDP markers among the evaluated lines. Among the genotypes, TAL105 exhibited superior heat tolerance, combining high productivity with strong biochemical and molecular defense mechanisms.

## Funding

Nottingham Trent University funded this research.

## Conflicts of Interest

The authors declare no conflicts of interest.

## Data Availability

The data that support the findings of this study are openly available on Zenodo at https://doi.org/10.5281/zenodo.22947014.
